# Binding of the Radioligand SIL23 to α-Synuclein Fibrils in Parkinson Disease Brain Tissue Establishes Feasibility and Screening Approaches for Developing a Parkinson Disease Imaging Agent

**DOI:** 10.1371/journal.pone.0055031

**Published:** 2013-02-06

**Authors:** Devika P. Bagchi, Lihai Yu, Joel S. Perlmutter, Jinbin Xu, Robert H. Mach, Zhude Tu, Paul T. Kotzbauer

**Affiliations:** 1 Department of Neurology, Washington University School of Medicine, St. Louis, Missouri, United States of America; 2 Department of Developmental Biology, Washington University School of Medicine, St. Louis, Missouri, United States of America; 3 Hope Center for Neurological Disorders, Washington University School of Medicine, St. Louis, Missouri, United States of America; 4 Department of Radiology, Washington University School of Medicine, St. Louis, Missouri, United States of America; 5 Department of Anatomy & Neurobiology, Washington University School of Medicine, St. Louis, Missouri, United States of America; 6 Program in Occupational Therapy, and Washington University School of Medicine, St. Louis, Missouri, United States of America; 7 Program in Physical Therapy, Washington University School of Medicine, St. Louis, Missouri, United States of America; Emory University, United States of America

## Abstract

Accumulation of α-synuclein (α-syn) fibrils in Lewy bodies and Lewy neurites is the pathological hallmark of Parkinson disease (PD). Ligands that bind α-syn fibrils could be utilized as imaging agents to improve the diagnosis of PD and to monitor disease progression. However, ligands for α-syn fibrils in PD brain tissue have not been previously identified and the feasibility of quantifying α-syn fibrils in brain tissue is unknown. We report the identification of the ^125^I-labeled α-syn radioligand SIL23. [^125^I]SIL23 binds α-syn fibrils in postmortem brain tissue from PD patients as well as an α-syn transgenic mouse model for PD. The density of SIL23 binding sites correlates with the level of fibrillar α-syn in PD brain tissue, and [^125^I]SIL23 binding site densities in brain tissue are sufficiently high to enable *in vivo* imaging with high affinity ligands. These results identify a SIL23 binding site on α-syn fibrils that is a feasible target for development of an α-syn imaging agent. The affinity of SIL23 for α-syn and its selectivity for α-syn versus Aβ and tau fibrils is not optimal for imaging fibrillar α-syn *in vivo*, but we show that SIL23 competitive binding assays can be used to screen additional ligands for suitable affinity and selectivity, which will accelerate the development of an α-syn imaging agent for PD.

## Introduction

Accumulation of misfolded, fibrillar α-syn in Lewy bodies (LB) and Lewy neurites (LN) is a definitive marker for the pathological diagnosis of PD. Pathological α-syn accumulation accompanies the degeneration of substantia nigra dopaminergic neurons underlying the motor features of PD, which include tremor, bradykinesia and rigidity. Approximately 80% of PD patients develop dementia within 20 years after onset of motor symptoms [1], and the development of cognitive impairment correlates with neocortical α-syn accumulation [2]–[4]. Since the clinical features of PD often overlap with those of other parkinsonian disorders, such as multiple system atrophy (MSA), progressive supranuclear palsy (PSP) and corticobasal degeneration (CBD), an imaging tracer that detects fibrillar α-syn accumulation could improve diagnostic accuracy for PD. Furthermore, quantifying the distribution of pathological α-syn *in vivo* could improve our understanding of disease progression, including the onset of dementia in PD patients. Thus, an imaging biomarker to quantify pathological α-syn could be valuable to test potential disease-modifying therapies and to distinguish more homogenous groups for therapeutic trials and treatment.

α-Syn is a presynaptic protein that normally exists in a natively unfolded state and is highly soluble [5], [6]. In PD, α-syn forms highly ordered insoluble aggregates called amyloid fibrils, which are stabilized by beta sheet protein structure [7], [8]. Fibrillar aggregate accumulation also occurs in other neurodegenerative diseases. Alzheimer’s disease (AD) is defined by accumulation of both amyloid Aβ and tau proteins. Pathological accumulation of tau but not Aβ occurs in a subset of frontotemporal lobar dementias [9]. Pittsburgh Compound B (PiB), a derivative of Thioflavin-T (ThioT), has been highly useful as a positron emission tomography (PET) probe for selectively quantifying amyloid Aβ *in vivo* [10]. PiB has high affinity for Aβ fibrils and for pathological Aβ in postmortem brain tissue [11]. PiB has also been reported to bind recombinant α-syn fibrils, but does not bind pathological α-syn in brain tissue and does not detect LB pathology when used as a PET probe [12]–[14]. Additional imaging ligands for Aβ fibrils and for tau fibrils are under development [15]–[18], but radioligands which bind fibrillar α-syn in PD brain tissue have not been previously reported.

We utilized binding studies with recombinant α-syn fibrils and preparations from postmortem brain tissue to identify and characterize a radioligand binding site on α-syn fibrils in PD. These studies are important to determine the feasibility of targeting α-syn fibrils *in vivo* for development of an imaging agent, and to establish methods for developing α-syn imaging ligands with suitable affinity and selectivity properties.

## Methods

### Ethics Statement

Because this research project only involved the use of postmortem tissue, it did not meet the definition of human subjects research and did not require approval by the Human Research Protection Office at Washington University in St. Louis. Written consent for autopsy was obtained from the next of kin at the time of death.

This study was carried out in strict accordance with the recommendations in the Guide for the Care and Use of Laboratory Animals of the National Institutes of Health. All animal procedures were performed according to protocols approved by the Washington University Animal Studies Committee (Protocol Number: 20110018).

### General

All reagents were purchased from Sigma-Aldrich (St. Louis, MO) unless otherwise indicated.

### Postmortem Human Brain Tissue Collection, Characterization

Brain tissue samples were selected from an autopsy case series of patients evaluated for parkinsonism by movement disorders specialists at the Movement Disorders Center of Washington University School of Medicine in St. Louis. The clinical diagnosis of idiopathic PD was based on modified United Kingdom Parkinson’s Disease Society Brain Bank clinical diagnostic criteria with clear clinical response to levodopa [19]. Dementia was determined by a movement disorders specialist based on clinical assessment of cognitive dysfunction sufficiently severe to impair activities of daily living, with further evaluation of cognitive impairment using the AD8 [20] and Mini-Mental Status Exam (MMSE) [21]. LB stage was assessed at autopsy using a PD staging scale (range: 0, 1–6) [4]. PD cases were selected based on a clinical diagnosis of PD plus dementia, Braak LB stage 5–6 pathology, and the absence of significant Aβ or tau pathology determined by immunohistochemistry. Control cases were selected based on the absence of α-syn, Aβ and tau pathology. Samples were used from both male and female subjects.

### Mice

Transgenic mouse lines expressing human A53T α-syn (M83 line) or human wild type α-syn (M7 line) were previously generated in the laboratory of Dr. Virginia M.Y. Lee (University of Pennsylvania) [22]. Mice used in this study were homozygous for each of the transgenes and were bred on mixed B6C3H and 129Sv backgrounds. Mice were housed and cared for in animal facilities administered through the Washington University Division of Comparative Medicine. All animal procedures were performed according to protocols approved by the Washington University Animal Studies Committee. M83 mice were observed for the development of neurological impairment and were euthanized after the onset of motor impairment. Brain tissue was removed and midbrain/pons/medulla tissue samples were dissected by first making a midsagittal cut using a brain matrix, which was then followed by an axial cut at the cervicomedullary junction and a second axial cut rostral to the superior colliculus for each hemisphere. Both male and female mice were used in the study.

### Radiosynthesis of [^125^I]SIL23

[^125^I]SIL23 was radiosynthesized by a halogen exchange reaction under the catalysis of Cu^+^ from the corresponding bromo-substituted precursor (SIL28). Two stock solutions were prepared for the radiolabelling: Solution A: ascorbic acid (116 mg) and SnSO_4_ (6 mg) dissolved in water (1 ml); Solution B: CuSO_4_·5H_2_O (2 mg) and 98% H_2_SO_4_ (60 µl) dissolved in water (2 ml). Both solutions were flushed with helium for 30 min. The precursor (1 mg) was dissolved in DMSO (300 µl) in a 2 ml reaction vessel containing a stir bar. Solution A (100 µl) and Solution B (200 µl) were added into the reaction vessel under nitrogen protection. The reaction vessel was sealed immediately after the addition of [^125^I]NaI (3 mCi) and heated at 130°C for 1 h. After the reaction mixture was cooled to room temperature, the reaction solution was diluted with 3 ml mobile phase and injected into a HPLC reverse phase semi-preparative column (Agilent SB-C18, 5 µm, 10×250 mm). The radioactive product was collected from 30 to 35 min on the HPLC condition (mobile phase: acetonitrile/water 60/40, v/v; flow rate: 4 ml/min; UV at 254 nm). The collected fraction was diluted with 40 ml of water and loaded on a C-18 Sep-Pak cartridge. After purification by HPLC again, the final product was eluted by ethanol (1 ml) to form the final solution (1.3 mCi, radiochemical yield 43%). Since [^125^I]SIL23 is effectively separated from the precursor on the semipreparative HPLC system described above and carrier-free [^125^I]NaI was used, it is assumed that the [^125^I]SIL23 is carrier-free with a theoretical specific activity of 2200 Ci/mmol [23]–[25].

### Preparation of Recombinant α-syn and Tau Protein

Recombinant protein was produced in E. Coli using protocols based on previously described methods for α-syn [26]–[28] and tau [29]. BL21(DE3)RIL E. Coli were transformed with a pRK172 bacterial expression plasmid containing the human α-syn coding sequence. Freshly transformed BL21 colonies were inoculated into 2 L baffled flasks containing 250 ml sterilized TB (1.2% bactotryptone, 2.4% yeast extract, 0.4% glycerol, 0.17 M KH_2_PO_4_, 0.72 M K_2_HPO_4_) with 50 µg/ml ampicillin, and incubated overnight at 37°C with shaking. Overnight cultures were centrifuged at 3,900×g for 10 min at 25°C and the bacterial pellets were resuspended by gentle vortexing in 20 ml osmotic shock buffer (30 mM Tris-HCl, 2 mM EDTA, 40% Sucrose, pH 7.2) and then incubated at room temperature for 10 min. The cell suspension was then centrifuged at 8,000×g for 10 min at 25°C and the pellet was resuspended in 22.5 ml cold H_2_O before adding 9.4 µl 2 M MgCl_2_ to each tube. The suspension was incubated on ice for 3 min prior to centrifugation at 20,000×g for 15 min at 4°C. After the supernatant was transferred to a fresh tube, streptomycin was added to a final concentration of 10 mg/ml and centrifuged at 20,000×g for 15 min at 4°C. The supernatant from this step was collected and dithiothreitol (DTT) and Tris-HCl pH 8.0 were added to final concentrations of 1 mM and 20 mM respectively, before boiling for 10 min to precipitate heat-sensitive proteins, which were pelleted at 20,000×g for 15 min at 4°C. The supernatant was collected and filtered through a 0.45 µm surfactant-free cellulose acetate filter (Corning) before loading onto a 1 ml DEAE Sepharose column equilibrated in 20 mM Tris-HCl pH 8.0, 1 mM EDTA, and 1 mM DTT. The DEAE column was washed with 20 mM Tris-HCl pH 8.0, 1 mM EDTA, 1 mM DTT before eluting α-syn protein in 20 mM Tris-HCl pH 8.0 buffer with 1 mM EDTA, 1 mM DTT and 0.3 M NaCl. Purified α-syn protein was dialyzed overnight in 10 mM Tris-HCl pH 7.6, 50 mM NaCl, 1 mM DTT. Preparations contained greater than 95% α-syn protein as determined by sodium dodecyl sulfate polyacrylamide gel electrophoresis (SDS-PAGE) and bicinchoninic acid (BCA) protein assay (Thermo Scientific, Rockford, IL), with a typical yield of 30 mg protein per 250 ml culture.

Recombinant tau protein was produced in E. Coli. BL21(DE3)RIL E. Coli were transformed with a pRK172 bacterial expression plasmid encoding a human tau fragment containing the four microtubule binding repeats (amino acids 243–375), provided by Marc Diamond at Washington University [30]. Cultures were inoculated and grown overnight as above for α-syn protein production. Purified tau protein was prepared using a previously described method [29] and dialyzed overnight in 100 mM sodium acetate pH 7.0.

### Preparation of Recombinant α-syn Fibrils

Purified recombinant α-syn monomer (2 mg/ml) was incubated in 20 mM Tris-HCl, pH 8.0, 100 mM NaCl for 72 h at 37°C with shaking at 1000 rpm in an Eppendorf Thermomixer. To determine the concentration of fibrils, the fibril reaction mix was centrifuged at 15,000×g for 15 min to separate fibrils from monomer. The concentration of α-syn monomer in the supernatant was determined in a BCA protein assay according to the manufacturer’s instructions, using a bovine serum albumin (BSA) standard curve. The measured decrease in α-syn monomer concentration was used to determine the concentration of fibrils in the 72 h fibril reaction mixture.

### Preparation of Aβ_1–42_ Fibrils

Synthetic Aβ_1–42_ peptide (1 mg) (Bachem, Torrance, CA) was first dissolved in 50 µl DMSO. An additional 925 µl of mQ-H_2_O was added. Finally, 25 µl 1M Tris-HCl pH 7.6 was added to bring the final peptide concentration to 222 µM (1mg/ml) [31]. The dissolved peptide was incubated for 30 h at 37°C with shaking at 1000 rpm in an Eppendorf Thermomixer. Fibril formation was confirmed by ThioT fluorescence. To determine the concentration of fibrils, the fibril reaction mix was centrifuged at 15,000×g for 15 min to separate fibrils from monomer. The concentration of Aβ monomer in the supernatant was determined in a BCA protein assay using a BSA standard curve that contained DMSO at a percentage equivalent to the samples.

### Preparation of Recombinant Tau Fibrils

Purified recombinant tau monomer (300 µg/ml) was incubated in 20 mM Tris-HCl pH 8.0, 100 mM NaCl, 25 µM low molecular weight heparin, 0.5 mM DTT for 48 h at 37°C with shaking at 1000 rpm in an Eppendorf Thermomixer. To determine the concentration of fibrils, the fibril reaction mixer was centrifuged at 15,000×g for 15 min to separate fibrils from monomer. The concentration of tau monomer in the supernatant was determined in a BCA protein assay along with a BSA standard curve. The measured decrease in monomer concentration was used to determine the concentration of tau fibrils in the 48 h fibril reaction mixture.

### Preparation of α-syn, Aβ_1–42_, and Tau Fibrils for Binding and Competition Assays

The prepared fibril mixture was centrifuged at 15,000×g for 15 min to prepare fibrils for binding assays. The supernatant was discarded and the fibril pellet was resuspended in 30 mM Tris-HCl pH 7.4, 0.1% BSA to achieve the desired concentration of fibrils for use in the assay.

### Preparation of Human Brain Tissue for *in vitro* Binding and Competition Studies

Grey matter was isolated from frozen postmortem frontal cortex tissue by dissection with a scalpel. To prepare insoluble fractions, dissected tissue was sequentially homogenized in four buffers (3 ml/g wet weight of tissue) with glass Dounce tissue grinders (Kimble): 1) High salt (HS) buffer: 50 mM Tris-HCl pH 7.5, 750 mM NaCl, 5 mM EDTA; 2) HS buffer with 1% Triton X-100; 3) HS buffer with 1% Triton X-100 and 1 M sucrose; and 4) phosphate buffered saline (PBS). Homogenates were centrifuged at 100,000×g after each homogenization step and the pellet was resuspended and homogenized in the next buffer in the sequence. For comparison in initial binding studies, crude tissue homogenates were also prepared by homogenization of tissue in only PBS.

### *In vitro* Saturation Binding Studies of [^125^I]SIL23

A fixed concentration (1 µM/well) of α-syn, Aβ, or tau fibrils were incubated for 2 h at 37°C with increasing concentrations of [^125^I]SIL23 (6.25–600 nM) in 30 mM Tris-HCl pH 7.4, 0.1% BSA in a reaction volume of 150 µl. A fixed ratio of hot:cold SIL23 was used for all radioligand concentrations. The exact hot:cold SIL23 ratio was measured in each experiment by counting a 10 µl sample of the radioligand preparation in a scintillation counter. Binding of [^125^I]SIL23 to human brain homogenates was assessed by incubating 10 µg samples of insoluble fraction or 50 µg of crude brain tissue homogenate, from PD-dementia or control subjects, with increasing concentrations of [^125^I]SIL23 (6.25–600 nM). Nonspecific binding was determined in a duplicate set of binding reactions containing the competitor ThioT. Bound and free radioligand were separated by vacuum filtration through 0.45 µm PVDF filters in 96-well filter plates (Millipore), followed by three 200 µl washes with cold assay buffer. Filters containing the bound ligand were mixed with 150 µl of Optiphase Supermix scintillation cocktail (PerkinElmer) and counted immediately. All data points were performed in triplicate. The dissociation constant (K_d_) and the maximal number of binding sites (B_max_) values were determined by fitting the data to the equation Y = B_max_*X/(X+K_d_) by nonlinear regression using Graphpad Prism software (version 4.0).

### *In vitro* Competition Studies of [^125^I]SIL23

Competition assays used a fixed concentration of fibrils (1 µM) or tissue (10 µg/150 µl reaction) and [^125^I]SIL23 (200 nM, consisting of a ratio of 1∶400 hot:cold SIL23) and varying concentration ranges of cold competitor, depending on the ligand. Competitors were diluted in 30 mM Tris-HCl pH 7.4, 0.1% BSA. Reactions were incubated at 37°C for 2 h before quantifying bound radioligand as described above for the saturation binding assay. All data points were performed in triplicate. Data were analyzed using Graphpad Prism software (version 4.0) to obtain EC_50_ values by fitting the data to the equation Y = bottom+(top-bottom)/(1+10^(x-logEC50)^). K_i_ values were calculated from EC_50_ values using the equation K_i_ = EC_50_/(1+[radioligand]/K_d_).

### Extraction of Insoluble α-syn for Western Blot and ELISA

Insoluble α-syn was isolated by sequential extraction of frozen postmortem human brain tissue as described previously [32]. Grey matter was isolated from frozen postmortem frontal cortex tissue by dissection with a scalpel. To prepare insoluble fractions for Western blot and ELISA analysis, dissected tissue was sequentially extracted in six buffers (3 ml/g wet weight of tissue) with glass Dounce tissue grinders (Kimble) [32]: 1, 2) High salt (HS) buffer: 50 mM Tris-HCl pH 7.5, 750 mM NaCl, 5 mM EDTA; 3) HS buffer with 1% Triton X-100; 4) HS buffer with 1% Triton X-100 and 1 M sucrose; and 5, 6) 1X radioimmunoprecipitation assay (RIPA) buffer. Extracts were centrifuged at 100,000×g after each step and the pellet was resuspended and extracted in the next buffer in the sequence. The final pellet was then resuspended in 50 mM Tris-HCl pH 8.0, 2% SDS (1 ml/g wet weight of tissue) and sonicated for 5 sec with 5 sec rest intervals in between for a total sonication time of 30 sec. Sonicated samples were centrifuged at 100,000×g and the supernatant was saved (SDS extract). The pellet was resuspended in 70% formic acid (1 ml/g wet weight of tissue) and sonicated for 5 sec with 5 sec rest intervals in between for a total sonication time of 30 sec. The formic acid was evaporated in a speed vacuum for 2 h. Then 1 volume of 50 mM Tris-HCl pH 8.0, 2% SDS was added to each sample to solubilize the protein. The samples were sonicated for 5 sec with 5 sec rest intervals in between for a total sonication time of 30 sec.

### Western Blot

Western Blot was performed as described previously [33]. Frontal cortex PD and control SDS extracts (8 µl) and anterior cingulate and temporal cortex PD extracts (4 µl) were run on an 18% Tris-glycine gel (Bio-Rad Criterion) and transferred to a nitrocellulose membrane as described previously [33]. The membrane was blocked with 5% nonfat milk in Tris buffered saline (TBS) with 0.1% Tween-20 for 1 h at room temperature, followed by incubation overnight at 4°C with syn1 (BD Biosciences) or syn303 [34], both mouse monoclonal antibodies against α-syn. The blot was then incubated with HRP-conjugated anti-mouse secondary antibody for 1 h at room temperature, followed by washing and detection with Immobilon enhanced chemiluminescence (ECL) reagent (Millipore). The blot was imaged with the G:Box Chemi XT4 (Synpotics) imager and was quantified using Multi-Gauge software (Fujifilm). Western blots included a standard curve of recombinant α-syn protein ranging from 2.5 ng to 30 ng. The ECL signal was linear over the range of the standards.

### Sandwich ELISA for α-syn

The levels of α-syn were measured by sandwich ELISA following the sequential extraction procedure. Mouse monoclonal α-synuclein 211 (Santa Cruz Biotechnology) was used as the capture antibody and biotinylated goat polyclonal anti-human synuclein-α (R&D Systems) was used as the detection antibody. PBS with 0.05%Tween 20, 2% BSA was used to block for 1 h at 37°C before adding samples. All washes were done in PBS-Tween 20. Bound detection antibody was quantified using Streptavidin Poly HRP80 (Fitzgerald) and SuperSlow 3,3′,5,5′-Tetramethylbenzidine (TMB) liquid substrate (Sigma-Aldrich). The standard curve was generated by combining bacterial recombinant α-syn with extracts prepared from control tissue samples, and ranged from 0 ng/well to 100 ng/well.

## Results

### [^125^I]SIL23 Binds to Recombinant α-syn Fibrils

**Figure 1:**
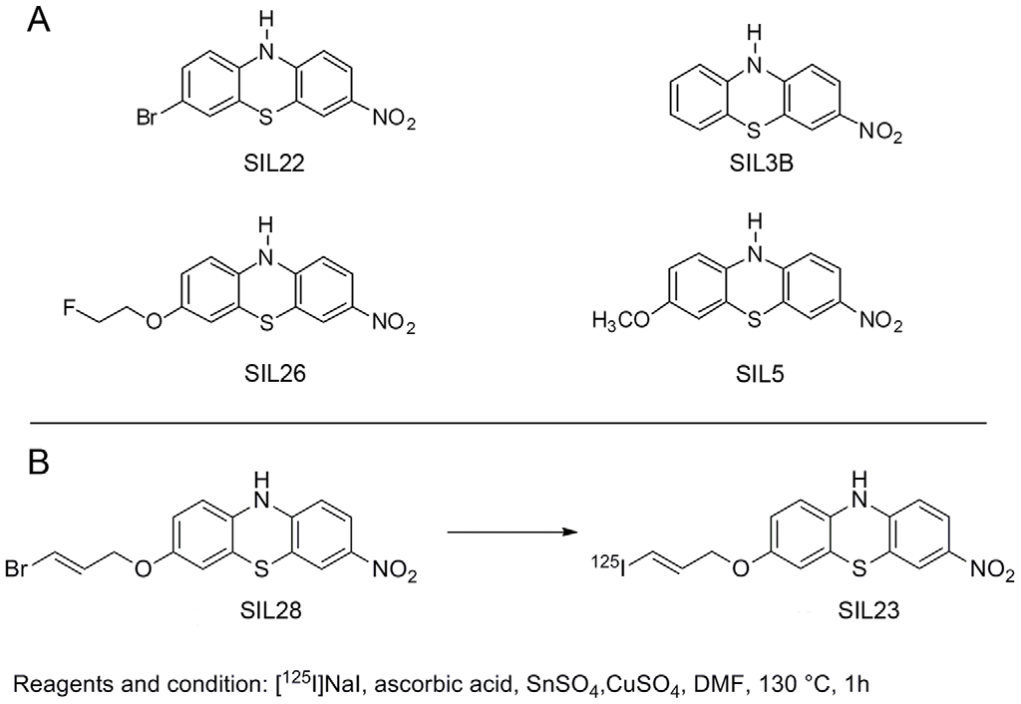
Phenothiazine structures and radiosynthesis of [^125^I]SIL23. Structures of SIL23 analogues are shown in **A**. The radiosynthesis of [^125^I]SIL23 is shown in **B**.

We previously utilized a fluorescent ThioT competition assay to identify a group of phenothiazine derivatives that bind fibrils prepared from recombinant α-syn protein (**Fig. 1A**) [28]. One of these compounds, (3-iodoallyl)oxy-phenothiazine (SIL23), displayed moderate affinity for α-syn fibrils (K_i_ 60 nM) in the ThioT competition assay, and was suitable for radiolabeling with ^125^I. Based on this result, we synthesized [^125^I]SIL23 to characterize the binding properties of this novel radioligand in fibril and tissue assays, and to determine its utility for screening additional compounds as candidate imaging ligands (**Fig. 1B**), which is an essential step in the development of an imaging agent for PD.

**Figure 2:**
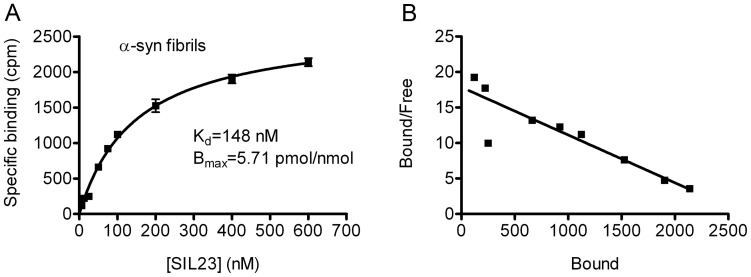
The radioligand [^125^I]SIL23 binds recombinant α-syn fibrils with a K_d_ of 148 nM. Fibrils prepared from recombinant α-syn were incubated with increasing concentrations of [^125^I]SIL23. Nonspecific binding was determined in parallel reactions utilizing 50 µM ThioT as competitor. A representative plot of specific binding versus [^125^I]SIL23 concentration is shown in **A**. Data points represent mean +/− s.d. (n = 3). The data was analyzed by curve fitting to a one-site binding model using nonlinear regression. The K_d_ value was determined by fitting the data to the equation Y = B_max_*X/(X+K_d_). Scatchard analysis of binding is shown in **B**. Similar results were obtained in more than three independent experiments.

We developed and optimized methods to measure the *in vitro* binding affinity of [^125^I]SIL23 to recombinant α-syn fibrils in saturation binding experiments. Recombinant α-syn fibrils were incubated with increasing concentrations of [^125^I]SIL23. We determined nonspecific binding in parallel reactions containing ThioT, unlabeled SIL23, or the phenothiazine analogue SIL5 as competitors, or in reactions containing radioligand but no fibrils, all of which yielded similar specific binding values. The binding data were analyzed by curve fitting using nonlinear regression to obtain K_d_ and B_max_ values. We observed specific binding of [^125^I]SIL23 to α-syn fibrils with a K_d_ of 148 nM and a B_max_ of 5.71 pmol/nmol α-syn monomer (**Fig. 2A**). We observed consistent binding values for five independently prepared fibril batches with K_d_ values ranging from 120 nM to 180 nM. Scatchard analysis indicates that the binding fits a one-site model (**Fig. 2B**).

**Figure 3:**
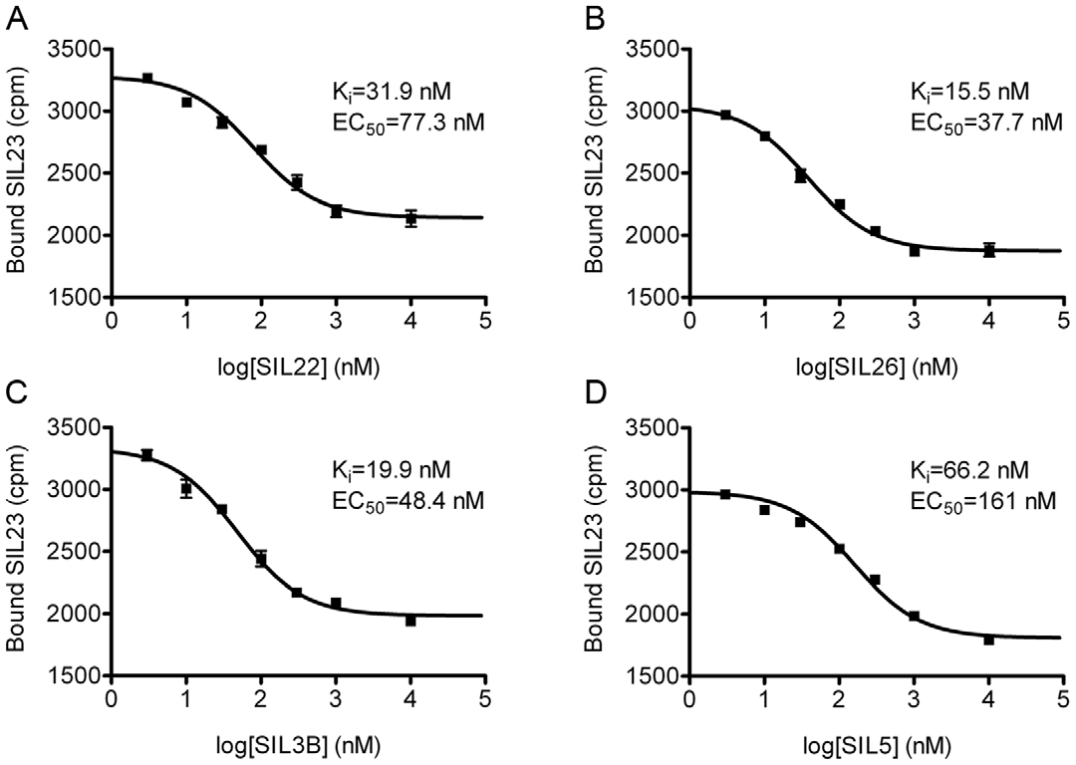
SIL23 competitive binding assays demonstrate higher affinity binding to recombinant α-syn fibrils for additional phenothiazine analogues, indicating that the structure of this compound class can be optimized to improve binding affinity using SIL23 assays. α-Syn fibrils were incubated with 200 nM [^125^I]SIL23 and increasing concentrations of SIL22 (**A**), SIL26 (**B**), SIL3B (**C**), and SIL5 (**D**). The amount of bound radioligand is plotted as a function of the concentration of unlabeled competitor ligand in the incubation mixture. Data points represent mean +/− s.d. (n = 3). EC_50_ values were determined by fitting the data to the equation Y = bottom+(top-bottom)/(1+10^(x-logEC50)^). Similar results were obtained in two independent experiments.

We developed a [^125^I]SIL23 competitive binding assay to enable the evaluation of binding affinities for additional phenothiazine analogues. Fixed concentrations of α-syn fibrils and [^125^I]SIL23 were incubated with increasing concentrations of each phenothiazine compound. Four analogues of [^125^I]SIL23, SIL22 (**Fig. 3A**), SIL26 (**Fig. 3B**), SIL3B (**Fig. 3C**), and SIL5 (**Fig. 3D**), were tested and had respective K_i_ values of 31.9 nM, 15.5 nM, 19.9 nM, and 66.2 nM, all with significantly higher affinities than the K_d_ for SIL23, indicating that SIL23 binding assays can guide the optimization of compound structures to increase binding affinity for α-syn fibrils.

### [^125^I]SIL23 and Additional SIL Analogues Exhibit Higher Binding Affinity to Recombinant α-syn Fibrils Compared to Synthetic Aβ_1–42_ or Recombinant Tau Fibrils

**Figure 4:**
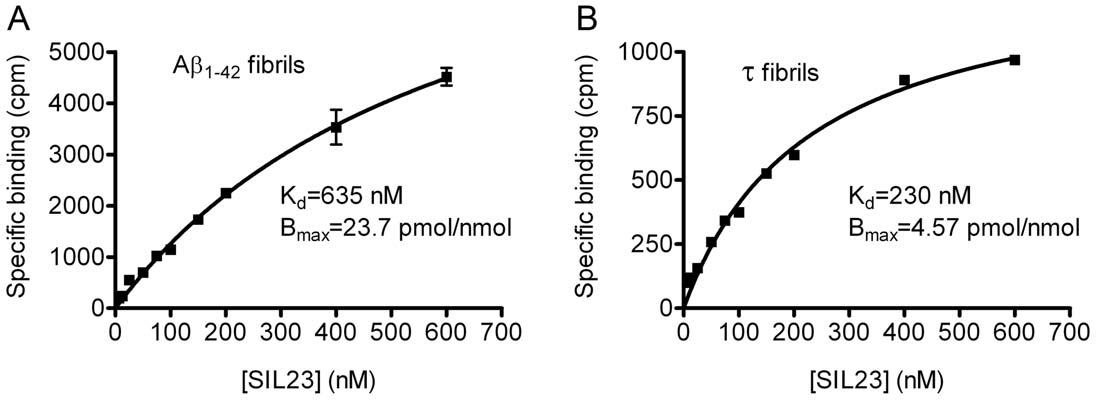
Radioligand binding studies demonstrate that [^125^I]SIL23 has selectivity for binding recombinant α-syn fibrils compared to synthetic Aβ_1–42_ fibrils or recombinant tau fibrils. Binding affinities of [^125^I]SIL23 to Aβ (**A**) and tau (**B**) fibrils were determined in saturation binding studies. The determined K_d_ values of Aβ and tau fibrils were 635 nM and 230 nM, respectively. Data points represent mean +/− s.d. (n = 3). Similar results were obtained in more than three independent experiments for saturation binding studies.

To determine the specificity of [^125^I]SIL23 for recombinant α-syn fibrils, we performed *in vitro* saturation binding studies on synthetic Aβ_1–42_ (**Fig. 4A**) and recombinant tau fibrils (**Fig. 4B**) and compared the results to data obtained from binding studies conducted on α-syn fibrils. Overall, the affinity of [^125^I]SIL23 for Aβ_1–42_ (K_d_ 635 nM, B_max_ 23.7 pmol/nmol) fibrils was 5-fold lower than that observed for α-syn fibrils. The affinity for tau fibrils (K_d_ 230 nM, B_max_ 4.57 pmol/nmol) was approximately 2-fold lower than α-syn fibrils.

To determine the specificity of other phenothiazine analogues for α-syn fibrils, we performed radioligand competition assays with Aβ_1–42_ (**Fig. S1A–D**) and tau fibrils (**Fig. S1E–H**) and compared the obtained K_i_ values to those obtained in radioligand competition assays with α-syn fibrils. All of the phenothiazine analogues examined in this study were selective for α-syn fibrils over Aβ_1–42_ and tau fibrils, but selectivity varied among analogues (**Table 1**). SIL26, which has the highest affinity for α-syn fibrils (K_i_ 15.5nM), has more than 6-fold lower affinity for Aβ_1–42_ (K_i_ 103 nM) and more than 7-fold lower affinity for tau fibrils (K_i_ 125 nM). These results obtained in SIL23 assays with Aβ_1–42_ and tau fibrils indicate that variations in phenothiazine structure can enhance selectivity as well as affinity for α-syn fibrils.

**Table 1:** Comparison of K_i_ values for SIL analogues in assays with α-syn, Aβ_1–42_, and tau fibrils illustrates relative selectivity for α-syn over Aβ_1–42_ and tau.

	*α-Syn fibrils*	*Aβ fibrils*	*Tau fibrils*
Competitor	K_i_ (nM)	K_i_ (nM)	K_i_ (nM)
SIL22	31.9 (22.1–45.9)	102 (87.3–119)	173 (144–208)
SIL26	15.5 (11.7–20.6)	103 (83.6–128)	125 (97.7–160)
SIL3B	19.9 (14.9–26.7)	71.5 (54.9–93.2)	52.3 (38.8–70.4)
SIL5	66.2 (49.2–89.1)	110 (94.7–127)	136 (112–165)

K_i_ values were calculated from EC_50_ values using the equation K_i_ = EC_50_/(1+[radioligand]/K_d_). 95% confidence intervals for K_i_ values are shown in parentheses.

### [^125^I]SIL23 Binds to Human PD Brain Homogenates

**Figure 5:**
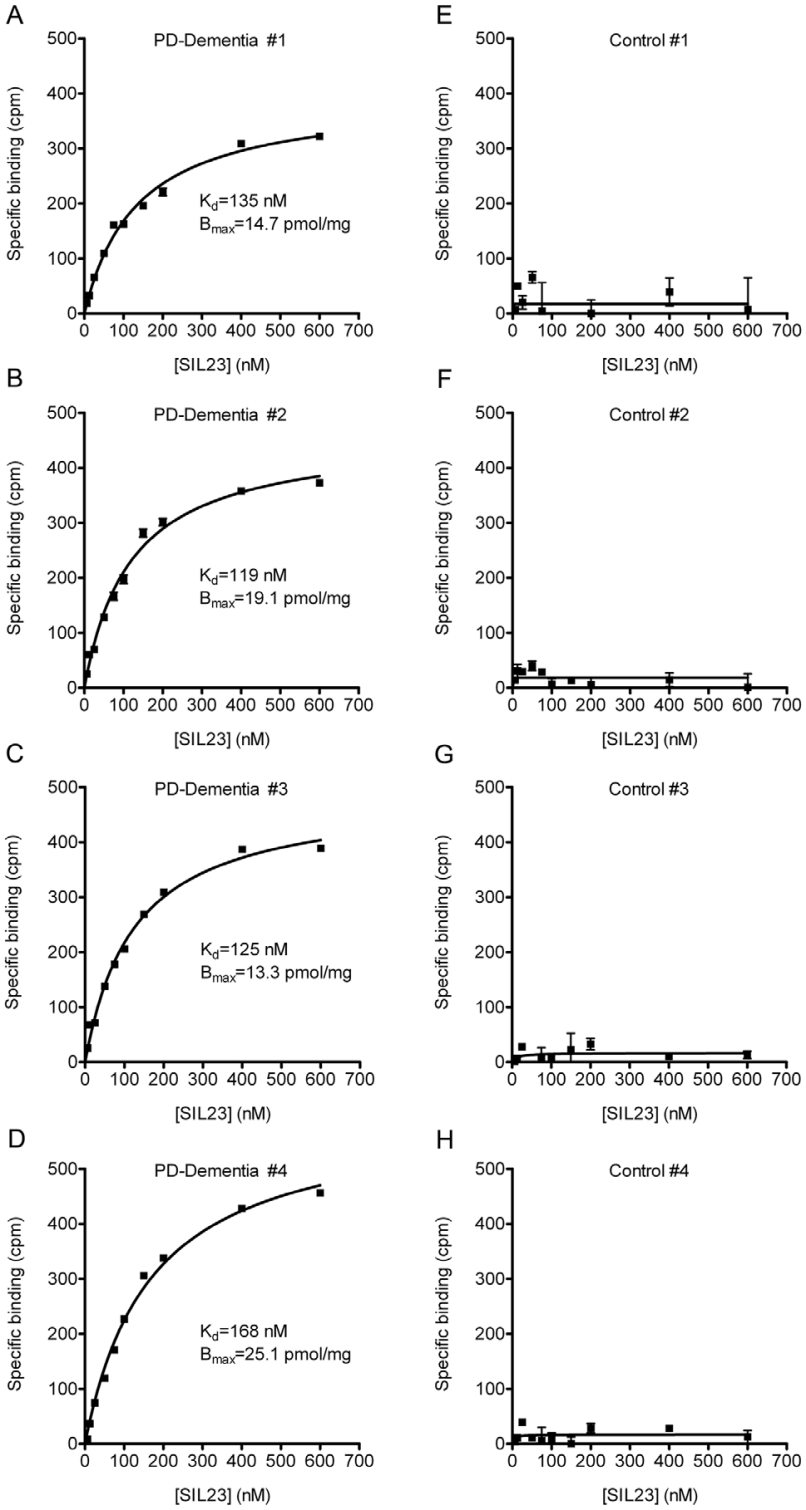
[^125^I]SIL23 exhibits specific binding to insoluble protein from human PD brain samples but not control human brain samples. Homogenized insoluble fractions from human brain samples (n = 8) were incubated with increasing concentrations of [^125^I]SIL23. Nonspecific binding was determined in parallel reactions utilizing 50 µM ThioT as competitor. Representative plots of specific binding versus [^125^I]SIL23 concentration are shown. **A–D** show four different PD cases and **E–H** show four control cases. The data was analyzed by curve fitting to a one-site binding model using nonlinear regression. K_d_ values for binding to PD-dementia brain samples range from 119.1 nM to 168.3 nM and B_max_ values range from 13–25 pmol/mg insoluble protein. No significant binding of [^125^I]SIL23 to control samples was observed. Results were verified with at least two independent experiments.

Previous studies have utilized binding assays with postmortem human brain homogenates to evaluate candidate amyloid imaging agents. Development of a similar assay with PD tissue is important to determine whether a binding site identified on recombinant α-syn fibrils is also present in PD tissue, and to determine whether the density of binding sites is high enough to image fibrillar α-syn *in vivo*. To evaluate [^125^I]SIL23 binding to fibrillar α-syn in LBs and LNs present in PD brain, we compared the *in vitro* binding of [^125^I]SIL23 in postmortem brain tissue from PD and control cases (**Table 2**), using insoluble fractions prepared from PD (n = 4) and control (n = 4) human brain tissue samples. K_d_ values for the PD cases ranged from 119 nM to 168 nM (B_max_ range 13.3–25.1 pmol/mg) (**Fig. 5A–D**). In contrast, we detected no significant [^125^I]SIL23 binding in the samples from the control cases (**Fig. 5E–H**). These results indicate that [^125^I]SIL23 binding affinity in PD brain samples is comparable to the binding affinity for recombinant α-syn fibrils.

**Table 2:** Clinical and demographic information for autopsy cases utilized for binding studies.

Case number	Age	Gender	Clinical Diagnosis	Pathologic findings
PD 1	69	M	PD, dementia	Diffuse Lewy body disease
PD 2	82	F	PD, dementia	Diffuse Lewy body disease
PD 3	78	M	PD, dementia	Diffuse Lewy body disease
PD 4	77	M	PD, dementia	Diffuse Lewy body disease
PD 5	79	M	PD, dementia	Diffuse Lewy body disease
C1	74	F	parkinsonism	Arteriosclerosis
C2	78	F	parkinsonism, dementia	Small vessel infarcts, argyrophilic grain disease
C3	85	M	parkinsonism	Argyrophilic grain disease, arteriosclerosis
C4	85	M	parkinsonism, dementia	Small and large vessel disease with neuronal loss

**Figure 6:**
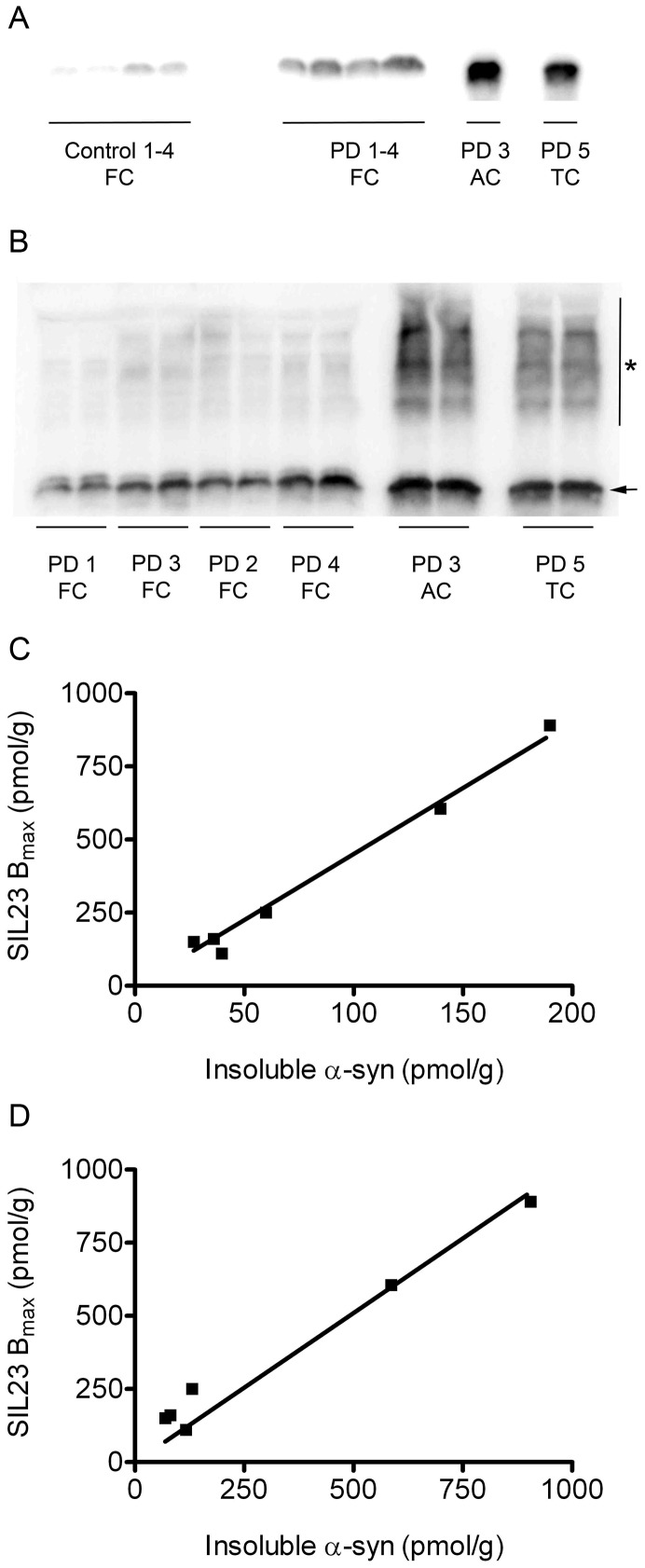
Binding of [^125^I]SIL23 is correlated with levels of insoluble α-syn present in PD brain. Levels of α-syn were measured in insoluble fractions from human brain samples (n = 10) by sequential extraction and western blot. A representative syn1 western blot with SDS extracts from PD and control cases is shown in **A**. A quantitative syn303 western blot with SDS extracts from PD cases is shown in **B**, in which higher molecular weight α-syn species (asterisk) are observed in combination with monomeric α-syn (arrow). The correlation of B_max_ values for [^125^I]SIL23 binding to levels of total insoluble α-syn quantified from the monomer band on western blot is shown in **C**. The correlation of B_max_ values for [^125^I]SIL23 binding to levels of total insoluble α-syn quantified from monomer plus high molecular weight species on western blot is shown in **D**. Results were verified with more than three independent experiments.

To determine whether SIL23 binding in different PD cases correlated with total levels of insoluble α-syn, we performed western blots on insoluble fractions prepared from PD (n = 6) and control (n = 4) human brain tissue samples. Western blot results showed that PD cases had different levels of insoluble α-syn, with anterior cingulate and temporal cortex PD samples showing the highest levels (**Fig. 6A**). In contrast, control cases had very low levels of detectable α-syn in insoluble fractions, which could represent low-level carryover of soluble α-syn during sequential extraction. In addition to monomeric α-syn, higher molecular weight species, likely representing multimeric α-syn, were also observed on western blots of insoluble fractions from PD cases (**Fig. 6B**). Insignificant levels of α-syn were observed by western blot analysis of formic acid extracts from the sequential extraction procedure (see methods).

Total monomeric α-syn present in insoluble SDS fractions quantified from western blot correlated with B_max_ values measured by the radioligand binding assay (Pearson correlation coefficient R = 0.99, p = 0.0001) (**Fig. 6C**). B_max_ values also correlated with insoluble α-syn measured by a sandwich ELISA (Pearson correlation coefficient R = 0.98, p = 0.0008) (**Fig. S2**). The ratios of B_max_ values to insoluble α-syn were approximately 4∶1. If multimeric species were included in the quantification of insoluble α-syn, the ratios of B_max_ values to insoluble α-syn were approximately 1∶1 (**Fig. 6D**) and were also correlated (Pearson correlation coefficient R = 0.99, p<0.001). Accuracies of these ratios may be limited by underestimation of insoluble α-syn due to low recovery during sequential extraction or incomplete solubilization of fibrils. A ratio of approximately 1 PiB binding site per 2 Aβ molecules has been observed in AD brain tissue [11].

*In vitro* [^125^I]SIL23 competition assays were used to evaluate the binding affinity of other SIL analogues with PD brain tissue samples (**Fig. S3A–D**). Fixed concentrations of homogenate and [^125^I]SIL23 were incubated with increasing concentrations of unlabeled competitor ligands. The K_i_ values obtained in assays with PD brain tissue homogenates were comparable overall to K_i_ values obtained with α-syn fibrils but were approximately 2-fold lower for some ligands (**Table 3**), indicating that SIL23 binding assays with recombinant α-syn fibril preparations accurately predict binding in tissue.

**Table 3:** Comparison of K_i_ values for SIL analogues in assays with recombinant α-syn fibrils versus human PD tissue.

	*α-syn fibrils*	*Human PD brain homogenate*
Competitor	K_i_ (nM)	K_i_ (nM)
SIL22	31.9 (22.1–45.9)	57.1 (44.9–72.6)
SIL26	15.5 (11.7–20.6)	33.5 (26.5–42.3)
SIL3B	19.9 (14.9–26.7)	49.4 (37.6–65.0)
SIL5	66.2 (49.2–89.1)	83.1 (64.3–108)

K_i_ values were calculated from EC_50_ values using the equation K_i_ = EC_50_/(1+[radioligand]/K_d_). 95% confidence intervals for K_i_ values are shown in parentheses.

We also used the [^125^I]SIL23 competition assay to evaluate other compounds known to bind amyloid fibrils. Fixed concentrations of α-syn fibrils and [^125^I]SIL23 were incubated with increasing concentrations of PiB, ThioT, BF227, and Chrysamine G (**Table 4** **, Fig. S4A–D**). ThioT displayed a K_i_ of 1040 nM, which is comparable to the K_d_ measured for saturation binding of ThioT to α-syn fibrils [28]. K_i_ values for PiB, BF227, and Chrysamine G were 116 nM, 39.7 nM, and 432 nM respectively. Additionally, we used the [^125^I]SIL23 competition assay in PD tissue homogenates to evaluate the binding properties of these compounds (**Table 4** **, Fig. S4E–H**). We obtained K_i_ values comparable to those for recombinant α-syn fibrils, with the exception of BF227, which displayed weaker competition in PD tissue assays, possibly corresponding to previous observations that radiolabeled BF227 binding is not detectable in PD tissue [35]. The K_i_ values for PiB and BF227 in the competition assays with α-syn fibrils were significantly higher than K_d_ values reported for the binding of radiolabeled PiB and BF227 to α-syn fibrils [13], [14], [35]. This could reflect differences in α-syn fibril preparations or may indicate that SIL23 binding sites only partially overlap with these previously reported ligands.

**Table 4:** K_i_ values of previously reported ligands for α-syn fibrils determined in [^125^I]SIL23 competitive binding assays with recombinant α-syn fibrils and PD tissue.

	*α-Syn fibrils*	*Human PD brain homogenate*
Competitor	K_i_ (nM)	K_i_ (nM)
PIB	116 (88.0–152)	99.2 (72.4–136)
BF227	39.7 (28.1–55.9)	138 (98.7–193)
Chrysamine G	432 (325–573)	367 (275–490)
Thioflavin T	1040 (755–1440)	974 (769–1230)

K_i_ values were calculated from EC_50_ values using the equation K_i_ = EC_50_/(1+[radioligand]/K_d_). 95% confidence intervals for K_i_ values are shown in parentheses.

We further evaluated binding site densities in PD brain using saturation binding assays with insoluble fractions from other cortical regions as well as binding assays performed with unfractionated homogenates of brain tissue samples. B_max_ values for insoluble fractions were significantly higher in temporal cortex and anterior cingulate cortex compared to frontal cortex (**Table 5**). For comparison to a previously reported average B_max_ value of 1407 pmol/g wet weight for PiB binding in AD brain [11], we estimated B_max_ values per gram wet weight for SIL23 binding in PD brain, which ranged from 8% to 63% of PiB values in AD (**Table 5**). Saturation binding studies using crude brain tissue homogenates rather than insoluble protein preparations also yielded a similar K_d_ value of 174 nM for a PD case while no significant binding was observed for a control case (**Fig. S5A–D**). The B_max_ value for the crude homogenate of a frontal cortex sample (PD 2) was 16.1 pmol/mg protein, which can be compared to an average B_max_ value of 8.8 pmol/mg protein observed in a similar assay for AV-45 binding in AD brain [17]. Nonspecific binding was significantly higher in binding assays with crude homogenates of brain tissue, possibly due to the high lipophilicity of SIL23 (calculated log P  = 5.7). High nonspecific binding, including nonspecific binding in white matter likely secondary to lipophilic interactions, also appears to limit autoradiography with SIL23 in preliminary experiments. Radioligands derived from other phenothiazine analogues with lower log P values [28] may overcome this issue.

**Table 5:** B_max_ values determined in saturation binding studies with PD brain tissue samples.

PD case	Cortical region	B_max_ (pmol/mg insoluble protein)	B_max_ (pmol/g wet weight)
1	midfrontal	14.7 (13.9–15.5)	145
2	midfrontal	19.1 (18.1–20.1)	160
3	midfrontal	13.3 (12.7–13.9)	108
4	midfrontal	25.1 (23.9–26.3)	251
5	temporal	53.1 (45.7–60.5)	607
3	anterior cingulate	91.1 (87.9–94.3)	895

B_max_ values are listed for frontal cortex from the four cases shown in **Figure 5**. B_max_ values were significantly higher in temporal cortex and anterior cingulate cortex samples. In the last column, B_max_ values from the same studies are expressed in pmol/g wet weight of brain tissue. 95% confidence intervals for K_i_ values are shown in parentheses.

### [^125^I]SIL23 Binding in a Transgenic Mouse Model for PD

**Figure 7:**
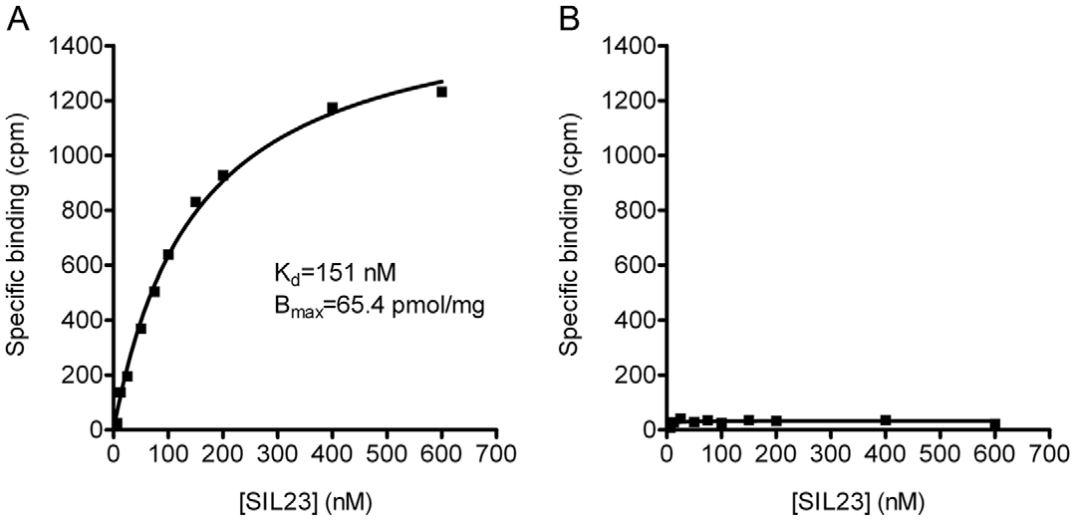
[^125^I]SIL23 binding sites are present at high density in a transgenic A53T α-syn mouse model for PD. [^125^I]SIL23 binding was determined in mouse brain samples obtained from (**A**) M83 mice with transgenic expression of human A53T α-syn, and (**B**) M7 mice with transgenic expression of human WT α-syn. Representative plots of specific binding versus [^125^I]SIL23 concentration are shown. The data was analyzed by curve fitting to a one-site binding model using nonlinear regression. [^125^I]SIL23 binding was detected in M83 brain samples with a K_d_ of 150.3 nM and a B_max_ of 65 pmol/mg insoluble protein. No significant [^125^I]SIL23 binding was detected in M7 brain. The results were verified with two independent experiments.

To determine whether SIL23 binding sites are also present in a transgenic mouse model for PD, we prepared brain tissue homogenates from transgenic mice expressing either a WT human α-syn transgene (M7 line) or a human α-syn transgene containing the A53T mutation that causes hereditary PD (M83 line) [22]. Accumulation of aggregated α-syn occurs primarily in brainstem and spinal cord of the M83 line but does not occur in the M7 line. M83 transgenic mice were observed and sacrificed when they displayed significant neurological impairment, which in this mouse line corresponds to the presence of aggregated α-syn in brain tissue. Tissue samples containing the midbrain, pons and medulla regions were dissected and processed by sequential extraction and centrifugation to prepare insoluble fractions. In saturation binding experiments, we observed specific binding of [^125^I]SIL23 in M83 tissue with a K_d_ of 151 nM and B_max_ of 65.4 pmol/mg (**Fig. 7A**). In contrast, we detected no significant [^125^I]SIL23 binding in M7 mouse brain homogenates (**Fig. 7B**). The K_d_ for binding in M83 tissue is similar to that observed for both recombinant α-syn fibrils and human brain tissue. The B_max_ value is comparable to B_max_ values observed for human cortex from PD cases. These results indicate that this A53T α-syn transgenic mouse model will be useful for evaluating *in vivo* binding of candidate α-syn imaging ligands, using micro-PET imaging or *ex vivo* autoradiography following radioligand injection.

## Discussion

Our results establish the presence of a [^125^I]SIL23 binding site on α-syn fibrils and define the binding properties of SIL23 in PD brain tissue. SIL23 binds with moderate affinity (K_d_ 148 nM) to α-syn fibrils. Furthermore, binding studies demonstrate that the fibrillar α-syn binding site is present in postmortem brain tissue from PD but not control cases and that binding site densities in PD tissue are comparable to binding site densities of Aβ imaging ligands in AD tissue. Competitive binding studies with [^125^I]SIL23 enable phenothiazine analogues as well as other compounds to be screened for affinity and selectivity for fibrillar α-syn. This is the first study to quantify and characterize a radioligand binding site for α-syn in PD brain tissue. The results demonstrate the feasibility of targeting α-syn fibrils for *in vivo* imaging approaches and establish approaches to identify ligands with suitable binding properties for imaging fibrillar α-syn *in vivo*. They also indicate that optimization of the structure of phenothiazine analogues could potentially achieve the desired affinity and selectivity required to quantify pathological α-syn *in vivo.*

An imaging tracer that enables quantification of fibrillar α-syn *in vivo* would improve the diagnostic accuracy in PD and could be highly useful as a biomarker for disease progression. Clinical diagnosis of PD currently relies on the presence of a combination of bradykinesia, rigidity, rest tremor and postural instability, plus the absence of additional features indicative of other parkinsonian disorders. Diagnostic challenges result from the fact that all features are not present in early PD and that these clinical features also occur in other disorders including vascular parkinsonism, MSA, CBD, and PSP. Parkinsonism is also a rare presenting feature in other neurodegenerative disorders, including frontotemporal dementias and AD. Rare hereditary neurodegenerative disorders can also present with parkinsonism, including syndromes categorized as hereditary parkinsonism, dystonia-parkinsonism, spinocerebellar ataxia, and neurodegeneration with brain iron accumulation. The accuracy of clinical diagnosis of idiopathic PD ranges from 76% to 92% [19], [36], [37] and is likely to be lower in early PD secondary to decreased sensitivity and specificity of the neurological exam.

A characteristic pattern of fibrillar α-syn deposition in LBs and LNs distinguishes PD from other disorders and is the defining feature for pathologic diagnosis. An imaging tracer that quantifies fibrillar α-syn *in vivo* would therefore greatly enhance the clinical diagnosis of PD. Amyloid deposits are absent in vascular parkinsonism and hereditary disorders such as spinocerebellar ataxia. Other parkinsonian disorders, including PSP and CBD, are defined by fibrillar tau accumulation. AD is defined by a combination of Aβ and tau accumulation. MSA, which has significant potential for phenotypic overlap with PD, is defined by the presence of fibrillar α-syn, but misfolded α-syn is instead deposited in glial cytoplasmic inclusions in a brain distribution pattern that is clearly distinct from PD, based on involvement of cerebellar and cerebral white matter, pontine nuclei in the basis pontis, and inferior olivary nuclei in the medulla [38]. This distinct distribution of pathology for MSA has also been seen in autopsy cases from patients early in the disease course and in asymptomatic individuals [39]–[41]. Spatial resolution of PET and SPECT scans may limit the ability to distinguish some brain regions such as pons and medulla that are differentially affected in PD and MSA, but resolution should be sufficient to identify MSA-specific pathology in regions such as cerebellar and cerebral white matter. Thus, the ability to identify the presence of fibrillar α-syn and to determine its distribution pattern in the brain could distinguish PD from most other disorders with overlapping clinical features, with the exception of rare hereditary disorders such as dystonia-parkinsonism caused by PLA2G6 mutations [42], [43]. Ultimately, clinical studies that include pathologic confirmation of diagnosis will be necessary to determine whether an α-syn imaging agent is useful for the differential diagnosis of parkinsonism. Given the presence of amyloid fibrils in other neurodegenerative disorders, *in vivo* selectivity for α-syn fibrils over tau and Aβ fibrils will be necessary for diagnostic accuracy.

Fibrillar α-syn imaging may also be a highly useful marker for disease progression. The distribution patterns for pathological α-syn among autopsy cases with incidental LB disease and symptomatic PD suggests that disease progression is associated with an ascending, progressive involvement of multiple brain regions. A staging system has been proposed [4], [44], in which early stage cases are defined by involvement of olfactory nucleus, as well as select nuclei in the medulla and pons. Intermediate stages are defined by additional involvement of substantia nigra pars compacta, basal forebrain cholinergic nuclei, and select nuclei within the hypothalamus and amygdala. Late stages are defined by progressive involvement of neocortex. The link between disease progression and increasingly widespread involvement of multiple brain regions is supported by the association of neocortical LBs with the development of dementia in PD, which occurs in up to 80% of patients within 20 years after onset of motor symptoms [1]. Longitudinal studies with an imaging tracer to quantify the amount and distribution of fibrillar α-syn *in vivo* would better define the natural disease course. This approach could also define the relative vulnerability of brain regions within the context of disease duration and establish correlations between the distribution of α-syn deposition and non-motor features of PD.

Accurate quantification of fibrillar α-syn *in vivo* will require a radiotracer with suitable affinity, selectivity, brain uptake and metabolism properties. Binding site density in brain is a critical factor in determining sensitivity and specificity for a radiotracer. We observe binding site densities of 110–890 pmol/g wet weight, and 16 pmol/mg protein in binding assays with crude homogenates, for SIL23 binding in human postmortem cortex. These values are comparable to binding site densities observed for Aβ ligands in AD brain. The average PiB binding site density is 1407 pmol/g wet weight in AD brain [11], and the average AV-45 binding site density is 8.8 pmol/mg protein in AD brain [17]. Based on K_d_ values of 2.5 nM for PiB and 3.7 nM for AV-45 binding to Aβ plaques, an α-syn binding site density of 16 pmol/mg protein, and an estimated brain concentration of 1 nM, a ligand that binds to the SIL23 binding site with a K_d_ of 7.3 nM could achieve binding to fibrillar α-syn in PD brain that is comparable to AV-45 binding in AD. Alternatively, a K_d_ of 2.5 nM, comparable to the K_d_ for PiB, will result in binding levels in cortex ranging from 8% to 63% of PiB levels.

Selectivity requirements for a candidate α-syn imaging ligand will be dictated by affinity, binding site density, and brain uptake. The fraction of binding sites occupied at a given ligand concentration is determined by the K_d_ based on [ligand]/([ligand]+K_d_). Assuming a brain concentration of 1 nM for an imaging tracer, selectivity in the range of 10- to 50-fold is desirable to achieve low binding to Aβ and tau fibrils. Selectivity could be further enhanced by the much higher dissociation rate for a binding site with 10- to 50-fold lower affinity. The relative densities of α-syn, Aβ and tau fibrils in PD and other neurodegenerative disorders causing parkinsonism is currently unknown. Our current estimates of selectivity are based on binding studies utilizing fibrils prepared from recombinant protein or synthetic peptide.

Evaluation of selectivity could be further improved by developing tissue binding assays with cases that are clinically relevant to the diagnosis of PD, including PSP, CBD, and AD. Since SIL23 is only moderately selective for α-syn fibrils over Aβ and tau fibrils, it is likely to have significant binding in AD, PSP and CBD tissue, and thus could be utilized in competitive binding assays to determine the selectivity of unlabeled ligands in tissue assays. However, given the combination of molecular pathologies often present in AD and other neurodegenerative disorders, this approach will require careful selection of cases and quantitative evaluation of α-syn, tau and Aβ pathology in each case. For example, selectivity evaluation could be enhanced by a panel of cases that includes 1) PD with dementia containing representative levels of α-syn pathology but no Aβ and tau pathology, 2) AD containing representative levels of Aβ and tau but no α-syn pathology, and 3) PSP/CBD containing only tau pathology. For high affinity ligands (K_d_<10 nM) suitable for radiolabeling, selectivity could be further evaluated in binding studies and autoradiography studies at radioligand concentrations comparable to those likely to be achieved *in vivo*.

Biomarkers for diagnosis and progression could accelerate the development of disease-modifying therapies in PD. Early stage PD may provide the greatest opportunity for effective intervention, yet accurate diagnosis is challenging. An effective diagnostic maker such as an α-syn imaging tracer would enable accurate enrollment of early stage PD patients into trials of therapeutic interventions targeting disease progression. If progressive accumulation of α-syn within individual regions or across multiple brain regions correlates with disease progression, particularly in early and intermediate disease stages, an α-syn imaging tracer could also greatly improve evaluation of therapeutic efficacy for candidate disease-modifying interventions. The development of SIL23 competitive binding assays is an important step in the development of a radiotracer for imaging α-syn aggregation *in vivo*. Since the SIL23 binding site on α-syn fibrils is a feasible radiotracer target, SIL23 competitive binding assay can be utilized to screen additional phenothiazine analogues as well as other classes of compounds to identify candidate imaging ligands with high affinity and selectivity for α-syn. Our results also demonstrate that SIL23 binding sites are present in a transgenic mouse model over-expressing A53T α-syn, indicating that brain uptake and *in vivo* binding of candidate α-syn imaging ligands can be evaluated in this mouse model with micro-PET or *ex vivo* autoradiography studies.

## Supporting Information

Figure S1 **Radioligand competition studies demonstrate that [^125^I]SIL23 analogues have selectivity for binding recombinant α-syn fibrils compared to synthetic Aβ_1-42_ fibrils or recombinant tau fibrils.** Competitive binding studies of Aβ and tau fibrils were performed with increasing concentrations of SIL22 (**A**, **E**), SIL26 **(B**, **F)**, SIL3B (**C**, **G**), and SIL5 (**D**, **H**). Data points represent mean +/− s.d. (n = 3). Similar results were obtained in more than two independent experiments. Nonspecific binding varied between individual experiments based primarily on washing times for individual filter plates, but the differences between top and bottom values in the competitive inhibition curves were similar among different compounds and between different experiments.
(TIF)

Figure S2 **Levels of α-syn were measured in insoluble fractions from human brain samples (n = 10) by sequential extraction and ELISA.** A representative plot of the correlation of B_max_ values for [^125^I]SIL23 binding to levels of total insoluble α-syn quantified from ELISA is shown (Pearson correlation coefficient R = 0.98, p = 0.0008). Results were verified with more than two independent experiments. Lower levels of α-syn measured in ELISA may result from low recovery during sequential extraction or incomplete solubilization of fibrils.
(TIF)

Figure S3 **Binding affinities for SIL analogues in human PD brain samples determined by competitive binding assays with [^125^I]SIL23.** Homogenized insoluble fractions from human PD brain samples were incubated with 200 nM [^125^I]SIL23 and increasing concentrations of competitor ligands. Representative plots are shown for competition with SIL22 (**A**), SIL26 (**B**), SIL3B (**C**), and SIL5 (**D**). The amount of bound radioligand is plotted as a function of the concentration of competitor ligand in the incubation mixture. The results were verified with two independent experiments.
(TIF)

Figure S4 **Binding affinities for previously reported α-syn ligands determined in [^125^I]SIL23 competitive binding assays with recombinant α-syn fibrils and PD tissue.** α-Syn fibrils (**A–D**) or insoluble fraction from human PD brain tissue (**E–H**) were incubated with 200 nM [^125^I]SIL23 and increasing concentrations of competitor ligands. Representative plots are shown for competition with ThioT (**A, E**), BF227 (**B, F**), chrysamine G (**C, G**), and PiB (**D, H**). The amount of bound radioligand is plotted as a function of the concentration of competitor ligand in the incubation mixture. The results were verified in two independent experiments.
(TIF)

Figure S5 **[^125^I]SIL23 exhibits similar specific binding to unfractionated human PD brain homogenates compared to insoluble protein fractions from human PD brain samples.** Crude PBS homogenates from human PD and control brain samples were incubated with increasing concentrations of [^125^I]SIL23. Nonspecific binding was determined in parallel reactions utilizing 50 µM ThioT as competitor. A representative plot of specific binding versus [^125^I]SIL23 concentration is shown for PD in **A** and for control in **C**. The data were analyzed by curve fitting to a one-site binding model using nonlinear regression. The K_d_ value for binding to PD-dementia crude brain homogenate was 174 nM, similar to K_d_ values obtained from insoluble fractions. The B_max_ value for finding to crude brain homogenate was 16.1 pmol/mg insoluble protein, which is within the range seen for insoluble fractions. No significant specific binding was observed for control brain. Results were verified with at least two independent experiments. Scatchard analysis of binding is shown in **B** for PD and **D** for control.
(TIF)
