# Supplementary figures and images for: Binding of the Radioligand SIL23 to α-Synuclein Fibrils in Parkinson Disease Brain Tissue Establishes Feasibility and Screening Approaches for Developing a Parkinson Disease Imaging Agent
Source: PLoS One. 2013 Feb 6;8(2):e55031. doi: 10.1371/journal.pone.0055031 (PMC3566091; doi:10.1371/journal.pone.0055031)

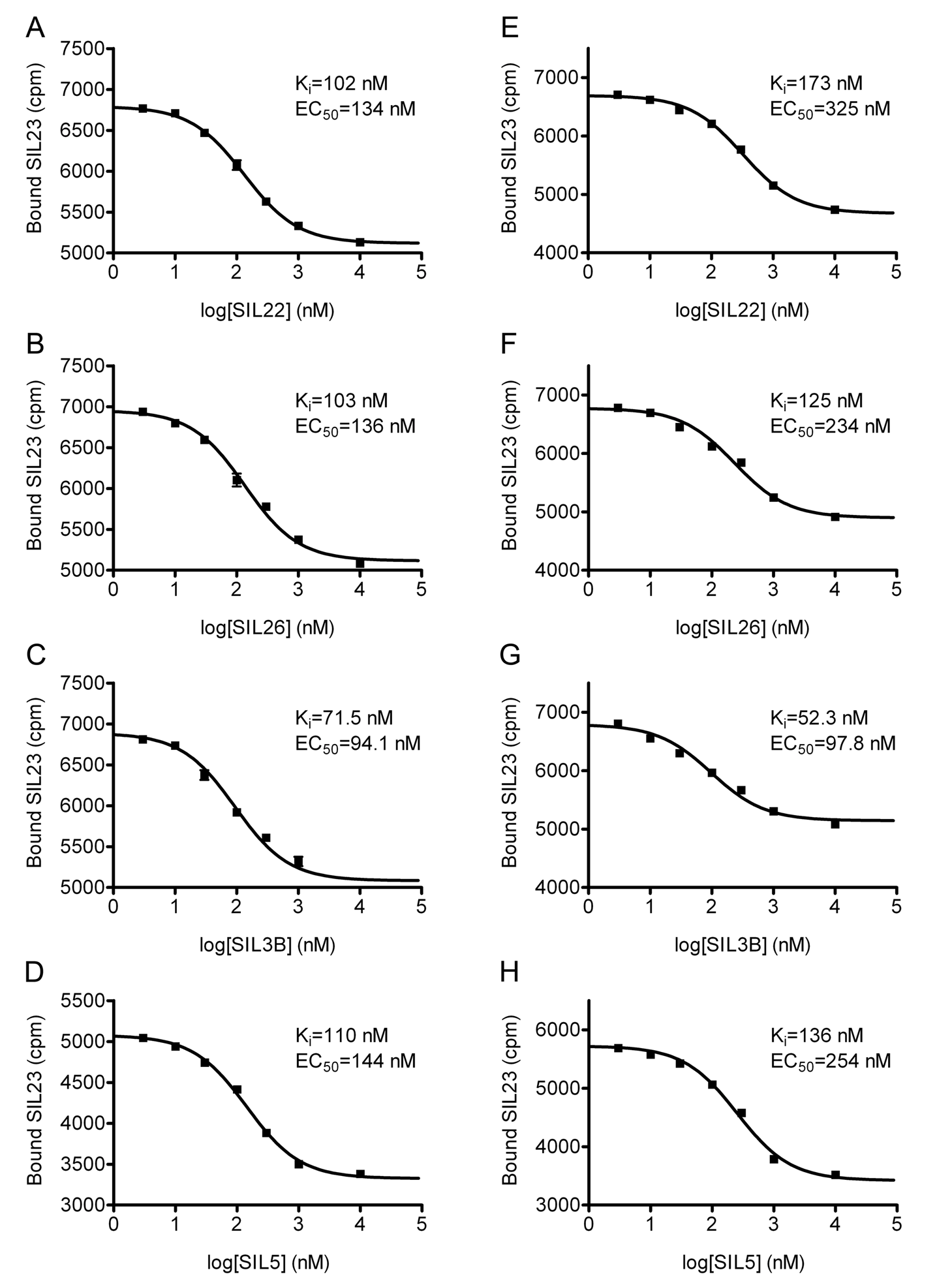

Supplement: Figure S1 — Radioligand competition studies demonstrate that [125I]SIL23 analogues have selectivity for binding recombinant α-syn fibrils compared to synthetic Aβ1-42 fibrils or recombinant tau fibrils. Competitive binding studies of Aβ and tau fibrils were performed with increasing concentrations of SIL22 (A, E), SIL26 (B, F), SIL3B (C, G), and SIL5 (D, H). Data points represent mean +/− s.d. (n = 3). Similar results were obtained in more than two independent experiments. Nonspecific binding varied between individual experiments based primarily on washing times for individual filter plates, but the differences between top and bottom values in the competitive inhibition curves were similar among different compounds and between different experiments. (TIF) [file pone.0055031.s001.tif]

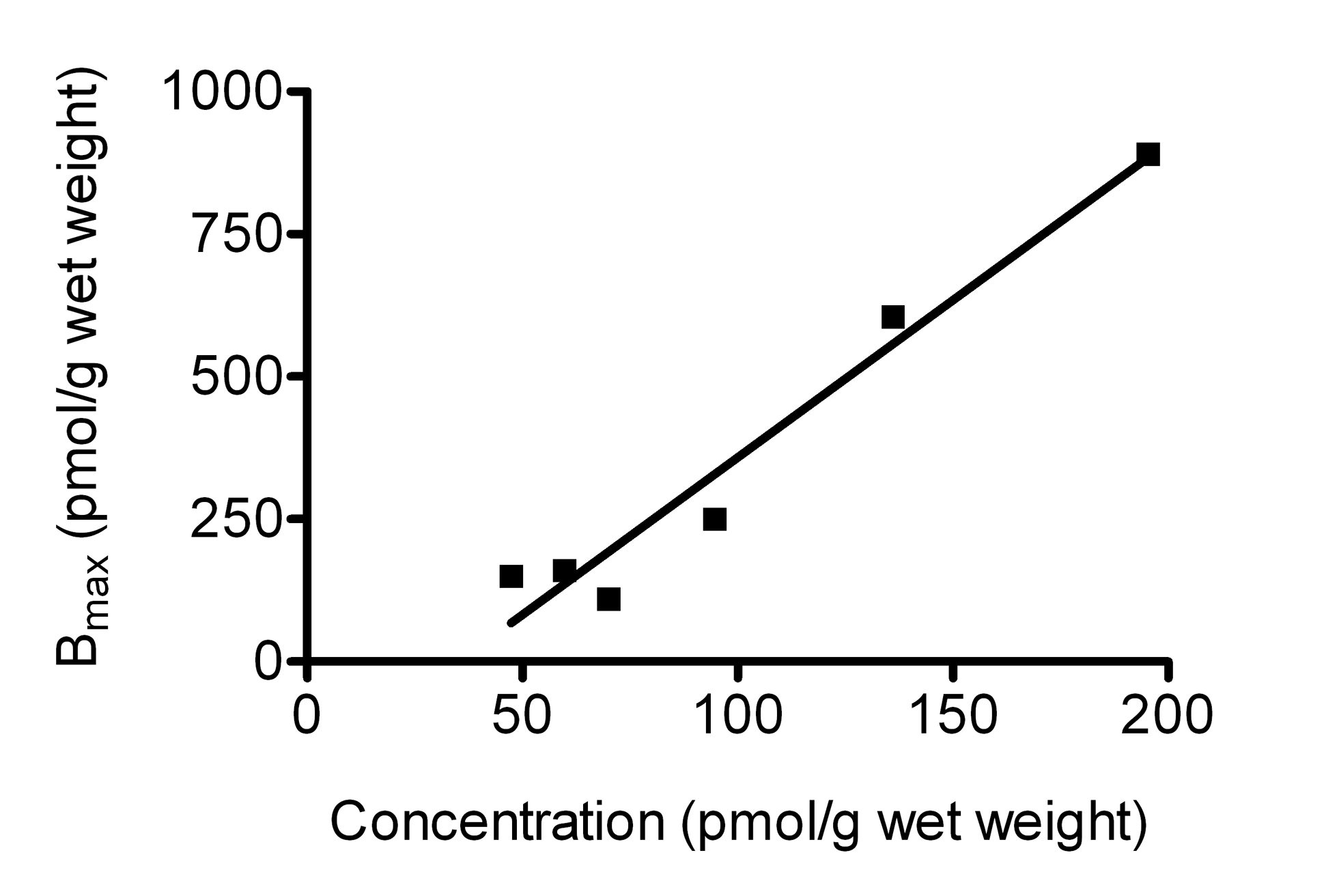

Supplement: Figure S2 — Levels of α-syn were measured in insoluble fractions from human brain samples (n = 10) by sequential extraction and ELISA. A representative plot of the correlation of Bmax values for [125I]SIL23 binding to levels of total insoluble α-syn quantified from ELISA is shown (Pearson correlation coefficient R = 0.98, p = 0.0008). Results were verified with more than two independent experiments. Lower levels of α-syn measured in ELISA may result from low recovery during sequential extraction or incomplete solubilization of fibrils. (TIF) [file pone.0055031.s002.tif]

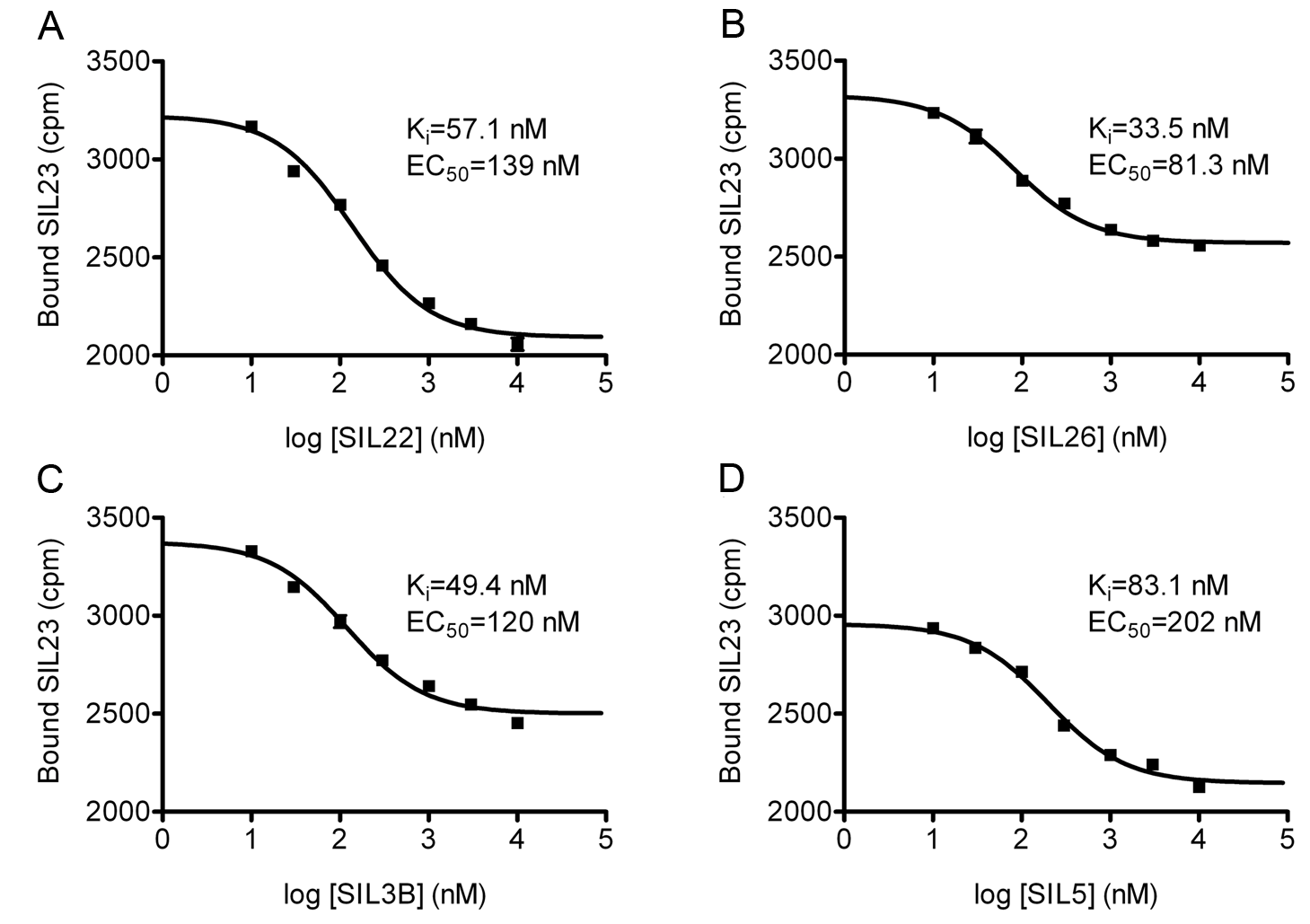

Supplement: Figure S3 — Binding affinities for SIL analogues in human PD brain samples determined by competitive binding assays with [125I]SIL23. Homogenized insoluble fractions from human PD brain samples were incubated with 200 nM [125I]SIL23 and increasing concentrations of competitor ligands. Representative plots are shown for competition with SIL22 (A), SIL26 (B), SIL3B (C), and SIL5 (D). The amount of bound radioligand is plotted as a function of the concentration of competitor ligand in the incubation mixture. The results were verified with two independent experiments. (TIF) [file pone.0055031.s003.tif]

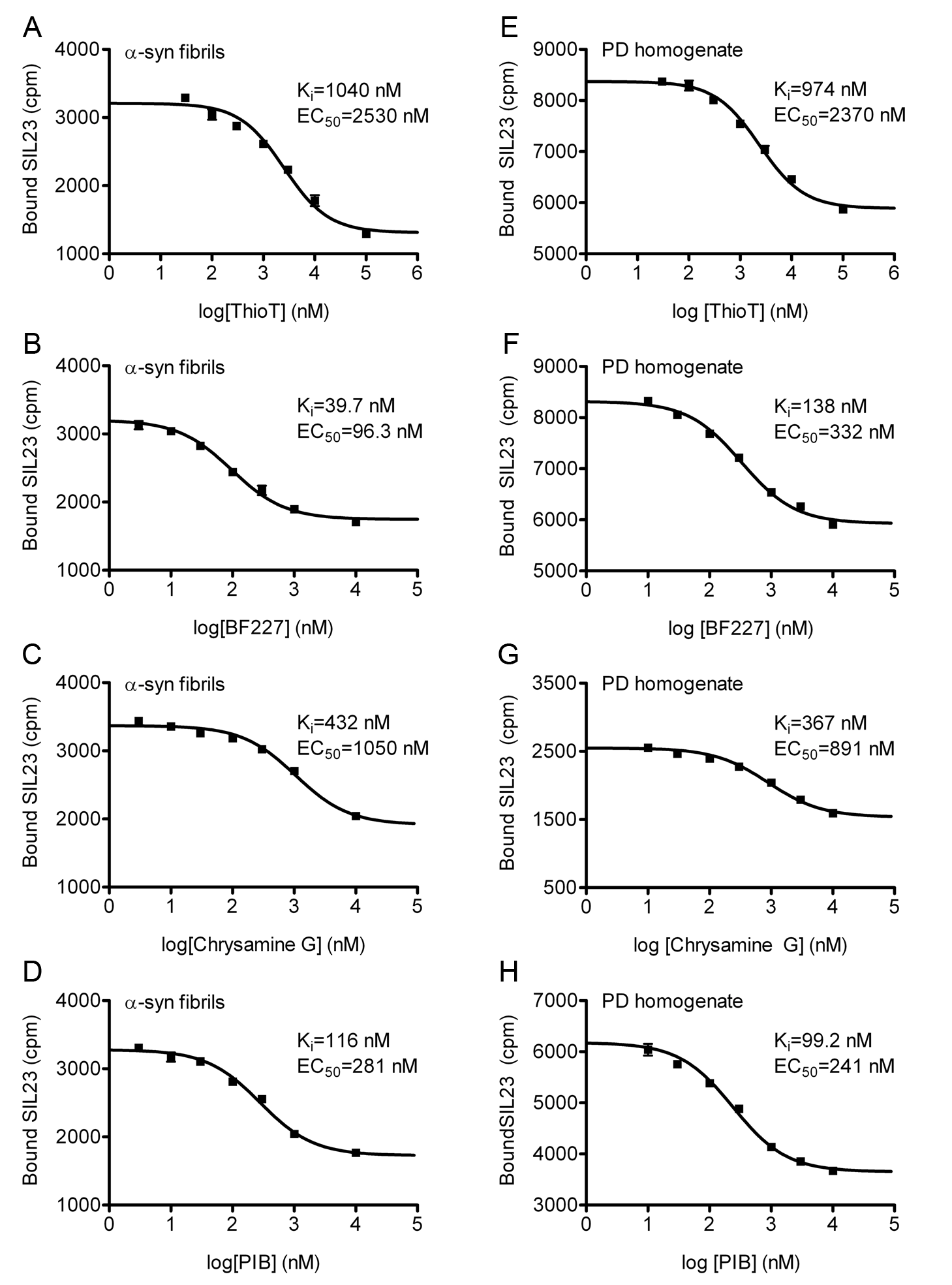

Supplement: Figure S4 — Binding affinities for previously reported α-syn ligands determined in [125I]SIL23 competitive binding assays with recombinant α-syn fibrils and PD tissue. α-Syn fibrils (A–D) or insoluble fraction from human PD brain tissue (E–H) were incubated with 200 nM [125I]SIL23 and increasing concentrations of competitor ligands. Representative plots are shown for competition with ThioT (A, E), BF227 (B, F), chrysamine G (C, G), and PiB (D, H). The amount of bound radioligand is plotted as a function of the concentration of competitor ligand in the incubation mixture. The results were verified in two independent experiments. (TIF) [file pone.0055031.s004.tif]

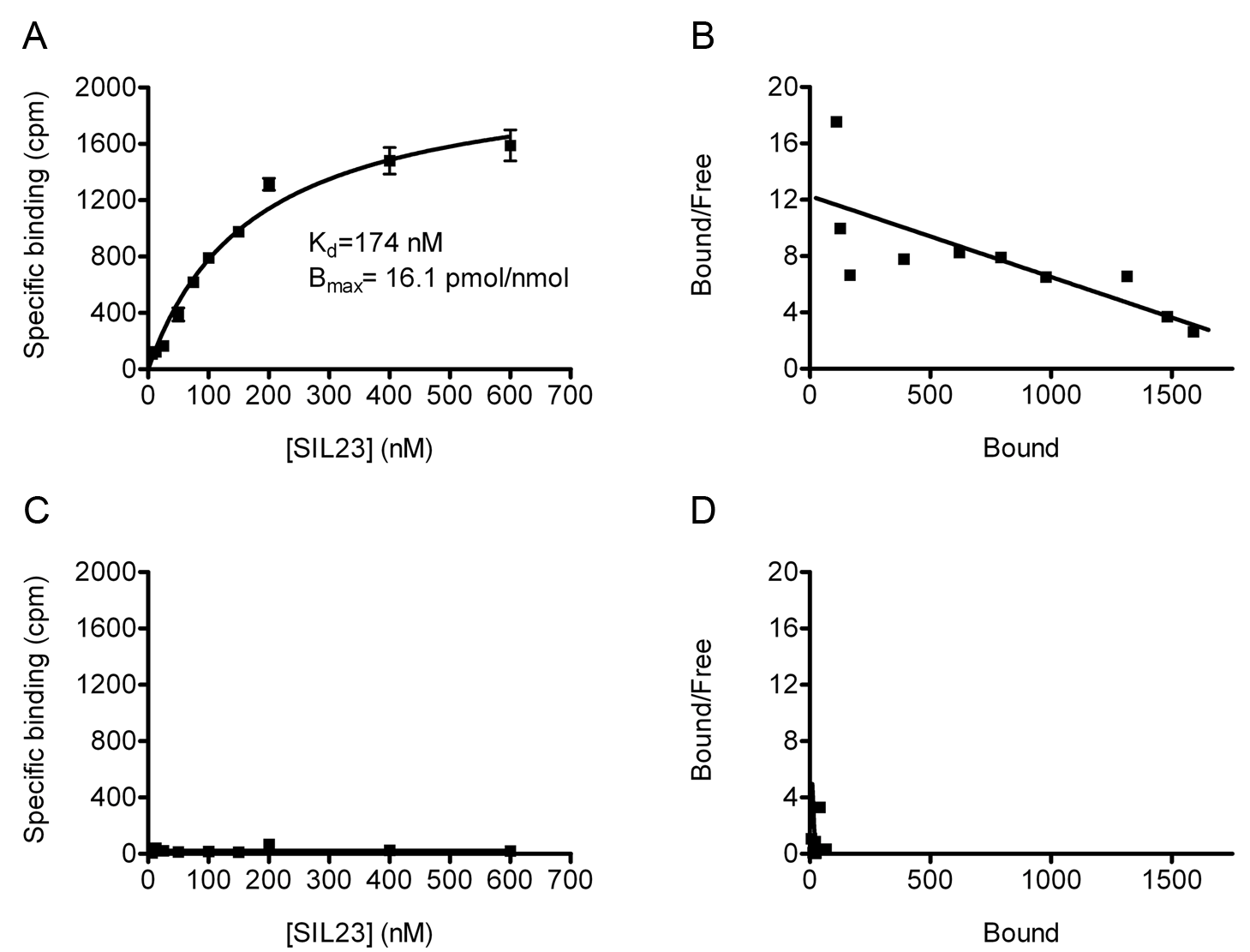

Supplement: Figure S5 — [125I]SIL23 exhibits similar specific binding to unfractionated human PD brain homogenates compared to insoluble protein fractions from human PD brain samples. Crude PBS homogenates from human PD and control brain samples were incubated with increasing concentrations of [125I]SIL23. Nonspecific binding was determined in parallel reactions utilizing 50 µM ThioT as competitor. A representative plot of specific binding versus [125I]SIL23 concentration is shown for PD in A and for control in C. The data were analyzed by curve fitting to a one-site binding model using nonlinear regression. The Kd value for binding to PD-dementia crude brain homogenate was 174 nM, similar to Kd values obtained from insoluble fractions. The Bmax value for finding to crude brain homogenate was 16.1 pmol/mg insoluble protein, which is within the range seen for insoluble fractions. No significant specific binding was observed for control brain. Results were verified with at least two independent experiments. Scatchard analysis of binding is shown in B for PD and D for control. (TIF) [file pone.0055031.s005.tif]
